# Sex differences in etiology and short-term outcome in young ischemic stroke patients receiving mechanical thrombectomy

**DOI:** 10.1186/s42466-022-00215-7

**Published:** 2022-10-17

**Authors:** Ralph Weber, Evgenia Winezki, Aristeidis H. Katsanos, Melissa Cueillette, Karim Hajjar, Elif Yamac, Roland Veltkamp, Rene Chapot

**Affiliations:** 1 Department of Neurology, Alfried Krupp Hospital Essen, Alfried-Krupp-Str. 21, 45131 Essen, Germany; 2 Division of Neurology, McMaster University & Population Health Research Institute, Hamilton, Canada; 3 Department of Brain Sciences, Imperial College London, London, United Kingdom; 4 Department of Neuroradiology, Alfried Krupp Hospital, Essen, Germany

**Keywords:** Ischemic stroke, Young age, Sex, Thrombectomy

## Abstract

**Background:**

Although there are well known sex differences in older patients with ischemic stroke receiving acute reperfusion treatments, there is paucity of data in younger patients.

**Methods:**

We investigated sex-related differences in clinical presentation, stroke etiology and short-term outcomes in consecutive young patients with acute ischemic stroke (AIS) below the age of 50 years receiving mechanical thrombectomy (MT) between January 2011 and May 2021 in a tertiary stroke center.

**Results:**

We identified a total of 202 young ischemic stroke patients with MT, with 51% being female. Young female AIS patients were significantly younger (39 ± 8 vs. 43 ± 7 years, p < 0.001), and presented with a trend for more severe stroke on admission (median NIHSS 12 vs. 9, p = 0.065), compared to males, respectively. Young female AIS patients had higher rates of embolic strokes of determined or undetermined sources in the anterior circulation, while young male AIS patients suffered more often strokes of arterio-arterial embolism. Complete reperfusion (TICI score 3) was achieved significantly less often in young female AIS patients (69% vs. 83%, p = 0.006), and in-hospital mortality was 2-times higher (5% vs. 2%, p = 0.271) compared to males.

**Conclusions:**

Young female AIS patients receiving MT have higher rates of severe embolic strokes and less often complete reperfusion due to different occlusion sites and stroke etiology compared to males.

## Introduction

Recent studies described a higher incidence of stroke in women, compared to men, before the age of 44 years [1]. Furthermore, large-scale nationwide and registry studies found a significantly higher rate of mechanical thrombectomy (MT) for large vessel occlusion (LVO) in women with acute ischemic stroke (AIS) irrespective of age [2, 3]. There are well known sex differences in baseline characteristics, stroke etiology and outcome in older patients with AIS receiving reperfusion treatments, with female AIS patients being older, having a higher rate of atrial fibrillation (AF) and increased stroke severity at presentation compared to men. However, the reason for the observed significantly increased MT rate in young female AIS patients from Germany is unknown [2, 3]. We therefore aimed to investigate sex-related differences in cardiovascular and non-cardiovascular risk factors, stroke etiology and short-term outcome in a large cohort of patients with AIS under the age of 50 years who were treated with MT.

## Methods

Hospital charts, laboratory values and imaging data of consecutive patients with AIS below the age of 50 years receiving MT between January 2011 and May 2021 in our tertiary stroke center were retrospectively reviewed for baseline characteristics, cardiovascular risk factors, and the following procedural and short-term outcome parameters: duration of digital subtraction angiography (DSA) for MT procedure, vessel recanalization at the end of the MT procedure, National Institute of Health Stroke Scale (NIHSS) score at 24 h and at discharge, symptomatic intracranial hemorrhage, and in-hospital mortality. Brain imaging, brain vessel imaging (CT- or MR-angiography, DSA, and extra-/intracranial ultrasound), extended electrocardiogram monitoring on the stroke unit or intensive care unit (at least 72 h in each patient), transthoracic or transesophageal echocardiography (with screening for patent foramen ovale [PFO]), laboratory testing were reviewed for all patients, and probable stroke etiology was determined by three authors (RW, EW, RC) by consensus. We regularly tested for the following thrombophilia if no other stroke etiology was found: Factor V Leiden mutation with testing for resistance to activated protein C, prothrombin G20210A mutation, Protein C or S deficiency, antiphospholipid syndrome (lupus anticoagulant and antiphospholipid IgM and IgG antibodies). We considered both pre-known and de-novo detected thrombophilia in our judgement of probable stroke etiology. Complete reperfusion after MT was defined as a Thrombolysis in Cerebral Infarction (TICI) score of 3, and was assessed on digital subtraction angiography imaging after the MT procedure. Symptomatic intracranial hemorrhage was defined according to the ECASS 3 definition [4].

Categorical data were presented with absolute numbers and corresponding percentages, while continuous data were reported in their mean values and corresponding standard deviations or median values with their respective interquartile ranges. Statistical comparisons between the two groups were performed using the chi-2 test, the t-test and the Mann–Whitney U-test, where appropriate. Analyses were performed with Stata Statistical Software Release 13 for Windows (College Station, TX, StataCorp LP).

## Results

From a total of 3555 patients with AIS receiving MT at our institution from January 2011 to May 2021, 202 patients were younger than 50 years, with 103 (51%) being female. Young female AIS patients were significantly younger (39 ± 8 vs. 43 ± 7 years; p < 0.001), and had a trend for more severe stroke syndromes on admission (median NIHSS 12 vs. 9; p = 0.065), due to significantly higher rates of occlusion of the internal carotid and middle cerebral artery, compared to males (Table 1). Female AIS patients had less frequently risk factors for atherosclerosis, with significant lower rates of arterial hypertension and diabetes, compared to males (Table 1). Nineteen of the 21 (86%) AIS patients classified to have suffered stroke due to PFO had a grade III PFO with substantial right-to-left shunting. One patient had additional atrial septum aneurysm.

**Table 1:** Comparison of patient characteristics, procedural and outcome parameters in young female and male acute ischemic stroke patients undergoing MT (all given percentages include missing values)

	Female patients	Male patients	p value
Number of patients	103	99	
Age, y, mean ± SD (min–max)	39 ± 8 (14–49)	43 ± 7 (19–49)	< 0.001
NIHSS score at admission, median (IQ range)	12 (7–18)	9 (4–16)	0.065
Bridging IVT, n (%)	53/102 (52%)	39/95 (41%)	0.125
Arterial hypertension, n (%)	36/102 (35%)	49/97 (51%)	0.030
Current smoker, n (%)	38/93 (41%)	51/93 (55%)	0.056
Diabetes mellitus, n (%)	5/92 (5%)	14/81 (17%)	0.013
Dyslipidemia, n (%)	36/98 (37%)	36/86 (42%)	0.477
Atrial fibrillation, n (%)	6/102 (6%)	3/97 (3%)	0.344
Substance abuse (e.g. cocaine), n (%)	1/102 (1%)	8/99 (8%)	0.015
Oral contraception use, n (%)	11/72 (15%)	N/A	N/A
Thombophilia, inherited or acquired, n (%)	16/100 (16%)	5/95 (5%)	0.016
*Occlusion site, n (%)*			
MCA	59/102 (58%)	41/98 (42%)	0.037
ICA	15/102 (14.5%)	10/98 (10%)	
VA	1/102 (1%)	5/98 (5%)	
BA	5/102 (5%)	14/98 (14%)	
PCA	2/102 (2%)	2/98 (2%)	
Multiple vessel occlusion	20/102 (19.5%)	26/98 (27%)	
Posterior circulation occlusion, n (%)	9/102 (9%)	28/98 (29%)	< 0.001
*Etiology, n (%)*			
Cardioembolic	10/103 (10%)	10/99 (10%)	0.001
LAA (≥ 50% stenosis)	9/103 (9%)	32/99 (32%)	
Arterial dissection	17/103 (16%)	19/99 (19%)	
Paradoxical embolism due to PFO	14/103 (13.5%)	7/99 (7%)	
Other determined etiology	13/103 (12.5%)	6/99 (6%)	
Undetermined etiology (ESUS)	38/103 (37%)	22/99 (22%)	
Two or more determined etiologies	2/103 (2%)	3/99 (3%)	
Duration of DSA for MT procedure, minutes, median (IQ range)	39 (20–66)	42 (25–66)	0.406
Complete reperfusion, TICI 3, n (%)	71/102 (70%)	82/97 (85%)	0.006
NIHSS score at 24 h, median (IQ range)	5 (1–12)	4 (1–11)	0.540
Symptomatic intracranial hemorrhage, n (%)	7/103 (7%)	3/99 (3%)	0.243
Hemicraniectomy, n (%)	11/103 (11%)	5/99 (5%)	0.072
NIHSS score at discharge, median (IQR range)	2 (0–7)	2 (0–5)	0.640
In-hospital mortality, n (%)	5/103 (5%)	2/99 (2%)	0.271
*Antithrombotic medication at discharge, n (%)*			
Antiplatelet	73/98 (74%)	79/97 (81%)	0.262
Oral Anticoagulation	23/98 (23%)	13/97 (13%)	
Both	1/98 (1%)	1/97 (1%)	

**Fig. 1:**
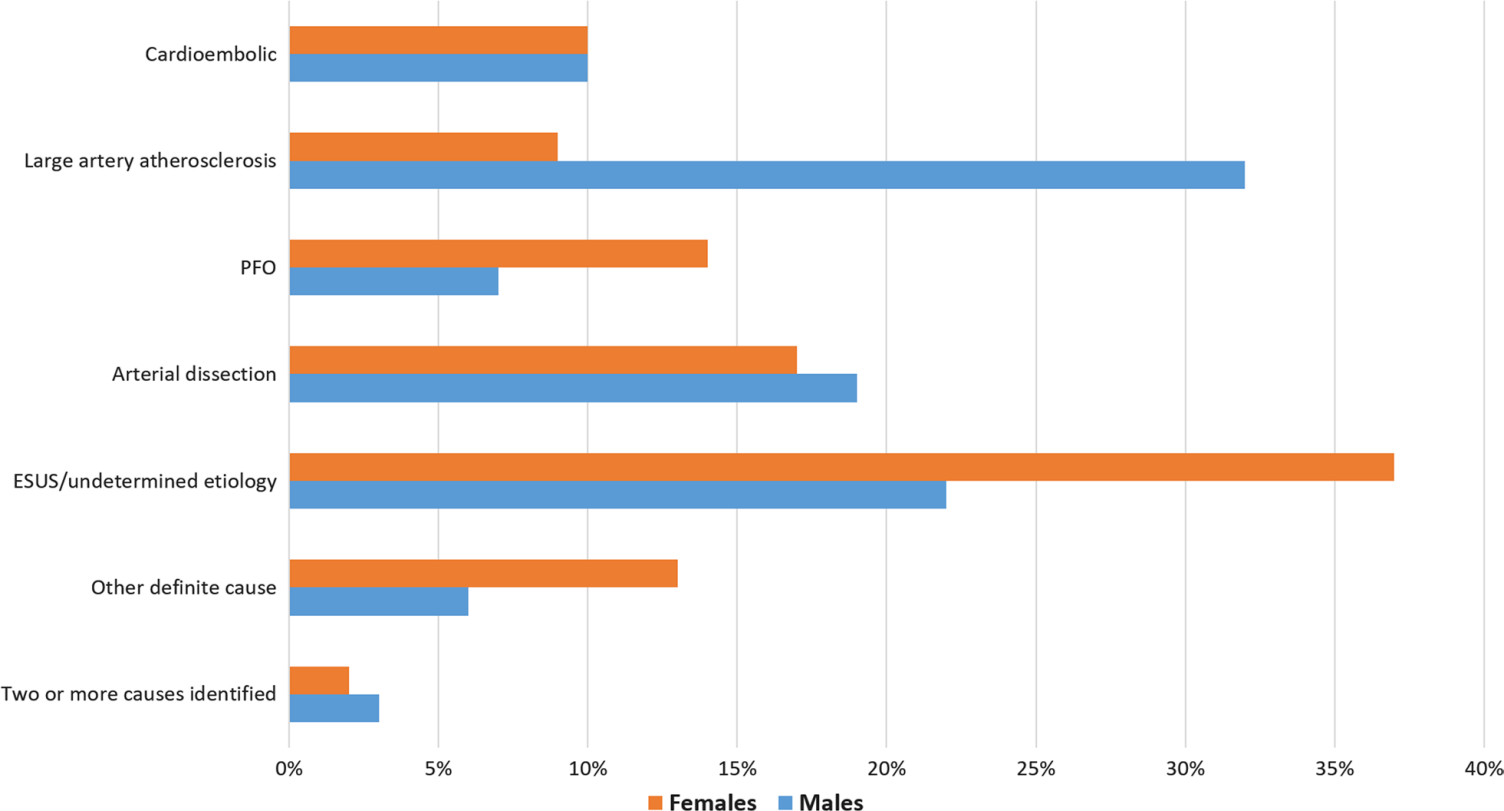
Causes of ischemic stroke in young female (orange) and male (blue) patients undergoing mechanical thrombectomy

There were significant sex differences in stroke etiology in our patient cohort. Young female AIS patients had higher rates of both embolic strokes of specified etiology (mostly PFO, thrombophilia or hypercoagulable states; Tables 1 and 2), and of undetermined source (ESUS), compared to males (Table 1, Fig. 1). Nine of the fourteen young female AIS patients classified with stroke due to a large PFO had additional findings suggestive of an underlying coagulation disorder (pregnancy-related, deep venous thrombosis, previous long-distance flight, known thrombophilia). In contrast, young male AIS patients had significantly more often arterio-arterial embolic strokes due to documented large artery atherosclerotic (LAA) with stenosis ≥ 50%. Other stroke etiologies including arterial dissection or AF did not differ between female and male AIS patients (Table 1).

**Table 2:** Other determined stroke causes in young female and male acute ischemic stroke patients

Young female ischemic stroke patients	Young male ischemic stroke patients
3 × Pulmonary shunts with thrombophilia	Carotid web
3 × perioperative stroke (+ thrombophilia in 2 patients)	Bow Hunter’s syndrome
2 × Gynecological malignancy with documented hypercoagulable state	Hepatitis C, heroin and cocaine intoxication
2 × Antiphospholipid syndrome	Thrombocytosis
Hodgkin’s Disease and thrombophilia	Active neurosyphilis with cerebral vasculitis
Factor V Leiden mutation with resistance to activated protein C and smoking	Partially thrombosed aneurysm of the basilar artery
Ovarian hyperstimulation syndrome

There was no significant difference in the median duration of the DSA for MT procedure between young female and male AIS patients (39 vs. 42 min, p = 0.406; Table 1). Complete reperfusion was significantly less often achieved in young female AIS patients compared to males (71/103; 69% vs. 82/99; 83%, p = 0.006), although young females received more often bridging IVT (52% vs. 41%, p = 0.125; Table 1). Twenty-two of the 32 young females with incomplete recanalization after MT had embolic strokes classified to be caused by PFO, hypercoagulable state, or ESUS, while the remaining had strokes due to CE, LAA and dissection. Although not significant, young female AIS patients experienced higher rates of symptomatic intracranial hemorrhage, hemicraniectomy and a more than two times higher rates of in-hospital mortality rate compared to males (5% vs. 2%, p = 0.271; Table 1).

## Discussion

We found significant sex differences in age presentation, risk factors, occlusion site and stroke etiology in a large cohort of young stroke patients with AIS treated with MT due to an LVO from a tertiary center specialized in neurointervention and evaluation/ treatment of stroke in young adults. Female AIS patients were significantly younger and had significant higher rates of embolic stroke in the anterior brain circulation due to determined (mainly associated with coagulation disorders and women-specific non-cardiovascular risk factors) or unknown embolic sources compared to males. Cardiovascular risk factors with LAA stenosis were significantly more prevalent in young male AIS patients, which was in line with an analysis of 1008 consecutive first-ever ischemic stroke patients aged 15 to 49 from the Helsinki Young Stroke Registry [5]. The rate of detected AF was low and did not differ between female and male AIS patients in our cohort. This is consistent with data from the Young ESUS longitudinal Cohort Study including 535 young adult patients with embolic stroke of undetermined source which also reported low rates of newly detected AF (2.8%) [6]. Arterial dissection was detected in 17.8% of our patient cohort, and did not significantly differ between young female and male AIS patients. This percentage lies within the range of 16% and 21.5% reported in AIS patients under 50 years of age undergoing MT in the MR CLEAN Registry Study and in a recently published multicenter cohort study, respectively [7, 8].

Leppert and colleagues suggested that risk factors, which are unique or more prevalent in women including pregnancy, oral contraception, migraine or autoimmune disorders could be an explanation for the increased stroke incidence in women aged 25 to 44 years [9]. In our cohort study, no AIS due to LVO was attributable to migraine or autoimmune disease, but female AIS patients suffered more often embolic strokes attributed to coagulation disorders and female sex hormone-related health issues. Despite a thoroughly performed diagnostic work-up, rate of ESUS was still considerable, and significantly higher in young female AIS patients undergoing MT compared to males in our study. The observed higher rate of embolic strokes due to determined and unknown sources in our study might explain why MT rates were significantly higher in younger female AIS patients compared to their male counterparts in a nationwide study in Germany [3].

Although young female AIS patients received slightly more often bridging IVT before the MT procedure, complete reperfusion was significantly less often achieved after the MT procedure in young females compared to young males. This bias might have led to an underestimation of reperfusion success in our study. Recanalization success was not caused by differences in the length of the MT procedure. One possible explanation for the significantly lower complete recanalization rate in young females with more embolic strokes due to causes other than LAA and arterial dissection might be difference in clot composition. Clots from CE stroke patients have been described to be more uniform, fibrin-rich. and hard to retrieve, while clots from artery-to-artery embolism consisted of a heterogeneous composition of fibrin and platelet aggregates, with higher red blood cell density [10]. Retrieved clots from patients with a hypercoagulable state had the lowest mean red blood cell density in the large retrospective Stroke Thromboembolism Registry of Imaging and Pathology (STRIP) [11]. Clots from ESUS patients had a significant lower red blood cell to platelet ratio in comparison with thrombi from stroke patients with LAA or carotid dissection in a small prospective study, sharing similar characteristics with CE thrombi [12].

Short-term NIHSS score did not differ between young female and male AIS patients at 24 h, but there was a trend towards higher rates of symptomatic intracranial hemorrhage and higher in-hospital mortality in young females in our study. Stroke patients with CE stroke undergoing MT had significantly longer puncture-to-recanalization times and lower rates of complete reperfusion compared to patients with LAA stroke in the retrospective study of Giray and co-workers [13]. A higher NIHSS score at baseline and 24 h in patients with CE stroke receiving IVT was also reported in the Helsinki Stroke Thrombolysis Registry compared to LAA [14]. Both aforementioned studies reported worse long-term functional outcome in CE stroke patients compared to LAA [13, 14]. Unfortunately, we are not able to provide long-term outcome data in our retrospective study, which is a major limitation of our study.

## Conclusions

In conclusion, our data suggest that young female AIS patients receiving MT have more severe embolic strokes in the anterior circulation due to more embolic stroke of determined and undetermined sources other than artery-to-artery embolism due to LAA, arterial dissection and cardioembolism, resulting in a lower rate of complete reperfusion.

## Data Availability

The anonymous dataset used and analysed during the current study are available from the corresponding author on reasonable request.

## Abbreviations

SD: Standard deviation
IVT: Intravenous thrombolysis
IQ: Interquartile
MCA: Middle cerebral artery
ICA: Internal carotid artery
BA: Basilar artery
VA: Vertebral artery
PCA: Posterior cerebral artery
LAA: Large artery atherosclerosis
IQR: Interquartile range
PFO: Patent foramen ovale
ESUS: Embolic stroke of unknown source
NIHSS: National Institutes of Health Stroke Scale
DSA: Digital subtraction angiography
TICI: Thrombolysis in cerebral ischemia
